# Monocyte-Containing Inflammatory Indices Show Stronger Association with 30-Day Mortality than the Systemic Immune-Inflammation Index in Elderly Sepsis: A Single-Center Retrospective Observational Cohort Study

**DOI:** 10.3390/jcm15124799

**Published:** 2026-06-20

**Authors:** Alexandru-Ionut Irimie, Sorin-Nicolae Dinescu, Marius-Bogdan Novac, Ramona-Constantina Vasile, Alexandra-Daniela Rotaru-Zavaleanu, Mihai-Andrei Ruscu, Lucretiu Radu

**Affiliations:** 1 Doctoral School, University of Medicine and Pharmacy of Craiova, 200349 Craiova, Romania; alexandru.irimie@umfcv.ro; 2 Department of Epidemiology, University of Medicine and Pharmacy of Craiova, 200349 Craiova, Romania; sorin.dinescu@umfcv.ro (S.-N.D.); alexandra.rotaru@umfcv.ro (A.-D.R.-Z.); mihai.ruscu@umfcv.ro (M.-A.R.); 3 Department of Anesthesiology and Intensive Care, University of Medicine and Pharmacy of Craiova, 200349 Craiova, Romania; 4 Department of Hygiene, University of Medicine and Pharmacy of Craiova, 200349 Craiova, Romania; lucretiu.radu@umfcv.ro

**Keywords:** sepsis, elderly, intensive care, AISI, SIRI, systemic immune-inflammation index, mortality, risk stratification, immunosenescence

## Abstract

**Background**. Hematological inflammatory indices from the complete blood count have been proposed as inexpensive prognostic markers in sepsis. The systemic immune-inflammation index (SII) and neutrophil-to-lymphocyte ratio (NLR) are the most studied, but the performance of monocyte-containing alternatives (SIRI, AISI) in the elderly, in whom immunosenescence may alter the leukocyte phenotype, remains poorly characterized. **Methods**. In a single-center retrospective cohort of patients aged ≥65 years admitted to a tertiary ICU with Sepsis-3-defined sepsis (*n* = 127, 33 deaths), we compared the discrimination of six indices (NLR, PLR, MLR, SII, SIRI, AISI) for 30-day all-cause mortality using AUROC with bootstrap confidence intervals and pairwise DeLong tests. Independent associations were assessed by logistic regression adjusted for APACHE II and age; incremental value over APACHE II was explored using IDI, cNRI, calibration and decision curve analysis, with bootstrap optimism correction. **Results**. Thirty-day mortality was 26.0%. The monocyte-containing indices (AISI, SIRI, MLR) discriminated better than SII and NLR, and AISI was significantly superior to SII, NLR and PLR on DeLong testing, though not to SIRI, MLR or APACHE II. After adjustment for APACHE II and age, AISI, SIRI and MLR remained independently associated with mortality, whereas SII and PLR did not. Adding AISI to APACHE II improved reclassification and calibration and yielded higher net clinical benefit across clinically relevant thresholds. **Conclusions**. In this exploratory, single-center analysis, monocyte-containing indices, particularly AISI, were more strongly associated with 30-day mortality in elderly ICU sepsis than SII or NLR. AISI, SIRI and MLR were strongly intercorrelated and near-equivalent, and AISI did not significantly exceed APACHE II in discrimination. These hypothesis-generating findings require prospective external validation before clinical use.

## 1. Introduction

Sepsis is defined as life-threatening organ dysfunction caused by a dysregulated host response to infection, according to the Third International Consensus Definitions (Sepsis-3) [1] which remain the prevailing international standard and continue to underpin the most recent Surviving Sepsis Campaign guidelines [2] and subsequent guideline updates [3]. Despite the consolidation of bundle-based care, hospital mortality of patients admitted to intensive care units (ICUs) with sepsis or septic shock remains in the range of 20–40% in contemporary cohorts and is highest at the extremes of age [4,5]. The elderly carry a disproportionate burden of sepsis: incidence rises sharply after the seventh decade, the proportion of culture-confirmed bacteremia is higher, comorbidities cluster, and the case-fatality rate is two- to threefold that of younger adults [6,7]. Risk stratification tools developed for the general ICU population (most prominently the Acute Physiology and Chronic Health Evaluation, APACHE II, and SOFA scores) perform reasonably well in older patients but were derived in mixed-age cohorts; their physiological inputs may underestimate the contribution of immune dysregulation, which is the proximate mechanism of organ failure in sepsis [8].

Over the past decade, indices derived from the differential complete blood count have been proposed as inexpensive, dynamically measurable surrogates of the systemic immune response. The neutrophil-to-lymphocyte ratio (NLR) and the platelet-to-lymphocyte ratio (PLR) were the first to be popularized, followed by the monocyte-to-lymphocyte ratio (MLR). The systemic immune-inflammation index (SII), introduced by Hu and colleagues in 2014 in hepatocellular carcinoma [9], integrates neutrophil, platelet and lymphocyte counts and has subsequently been examined in coronary disease, neoplasms and infection. The systemic inflammation response index (SIRI = neutrophils × monocytes/lymphocytes) and the aggregate index of systemic inflammation (AISI = neutrophils × monocytes × platelets/lymphocytes) are conceptually similar but explicitly incorporate monocytes, the principal cellular source of pro-inflammatory cytokines in early sepsis.

The largest study of these biomarkers in bloodstream infection (BSI), by Ou and colleagues in 469 patients [10], reported that SII (AUROC 0.760) and NLR (0.765) outperformed SIRI, AISI, PLR and MLR. That report is now widely cited as evidence that platelet- and neutrophil-driven indices are the optimal hematological prognosticators in BSI. There are, however, substantive reasons to doubt that this hierarchy generalizes to elderly ICU sepsis. First, Ou et al. enrolled an outpatient/inpatient mixed cohort with a median age below 65 in which 53% of subjects were younger than 65 [10]; their analyses did not adjust for severity of illness scores. Second, immunosenescence (the age-related contraction of the naïve T-cell pool, accumulation of terminally differentiated CD8 cells, and elevated baseline NLR even in the absence of infection) confers reduced specificity to lymphocyte-based ratios in older adults [11,12]. Third, monocytes show distinctive perturbations in elderly sepsis, including expansion of intermediate (CD14^++^CD16^+^) subsets, depressed monocyte HLA-DR expression, and increased monocyte distribution width (MDW), the latter recently endorsed by the FDA as a stand-alone diagnostic marker for sepsis [13,14]. These observations predict that indices incorporating monocytes (SIRI, AISI, MLR) should capture prognostic information that platelet-dominated indices (SII, PLR) and the simple NLR cannot.

We therefore examined the discriminatory and incremental prognostic value of six hematological inflammatory indices for 30-day mortality in a cohort of elderly patients admitted to a tertiary ICU with Sepsis-3-defined sepsis. We pre-specified four hypotheses: (i) AISI and SIRI would demonstrate higher AUROC for 30-day mortality than SII and NLR; (ii) the monocyte-containing indices would retain independent association with mortality after adjustment for APACHE II and age, whereas SII and NLR would not; (iii) the addition of AISI to APACHE II would yield clinically meaningful reclassification (IDI, cNRI) over the severity score alone; and (iv) a parsimonious two-marker model (APACHE II + AISI) would provide superior net clinical benefit on decision curve analysis across the threshold-probability range relevant to ICU triage.

## 2. Materials and Methods

### 2.1. Study Design and Setting

This was a single-center, retrospective observational cohort study conducted in a 12-bed mixed medical–surgical ICU of a tertiary university hospital in south-western Romania. The study was reviewed and approved by the local research ethics committee of Filantropia County Hospital (approval no. 30198/4 December 2025), and the requirement for individual informed consent was waived in accordance with national regulations governing the use of fully anonymized clinical data. The reporting follows the STROBE recommendations for observational research [15] and the TRIPOD statement for prognostic studies [16].

### 2.2. Participants

Consecutive adults (≥18 years) admitted to the ICU between January 2025 and November 2025 were screened. Sepsis was defined according to the Third International Consensus Definitions for Sepsis and Septic Shock (Sepsis-3) and the 2021 Surviving Sepsis Campaign guidelines [1,2] as documented or strongly suspected infection plus an acute increase in the SOFA score of ≥2 points attributable to that infection. Suspected infection was operationally defined as the prescription of empirical broad-spectrum antibiotics combined with collection of microbiological cultures within 48 h of admission. Patients with hematological malignancy on active chemotherapy, those on chronic immunosuppression equivalent to ≥10 mg prednisone daily, recipients of solid-organ transplant within 90 days, and patients with incomplete admission complete blood count were excluded. The pre-specified primary analytic cohort consisted of patients aged ≥65 years, since this group represents the population in which the operational characteristics of hematological indices are least understood and most likely to be affected by immunosenescence. Patients aged <65 years were retained for descriptive and exploratory comparisons but were not included in inferential analyses because the resulting event count (1 death in 42 patients) precluded meaningful estimation.

### 2.3. Variables and Inflammatory Indices

Demographic data, comorbid conditions, source of infection, mechanical ventilation requirement, length of ICU and hospital stay, and admission laboratory values were extracted from the electronic medical record. SOFA and APACHE II were calculated using the worst values within the first 24 h of ICU admission. Hematological indices were computed from the admission complete blood count using the standard formulae [9,10]:

NLR = absolute neutrophil count/absolute lymphocyte count

PLR = platelet count/absolute lymphocyte count

MLR = absolute monocyte count/absolute lymphocyte count

SII = (neutrophils × platelets)/lymphocytes

SIRI = (neutrophils × monocytes)/lymphocytes

AISI = (neutrophils × monocytes × platelets)/lymphocytes

All cell counts were expressed as ×10^9^/L. We elected to use admission values rather than pre-defined trajectories because the goal was to evaluate a single-time-point prognostic marker available at the moment of triage, when clinical decisions are most consequential.

The total number of comorbidities per patient was computed as the count of affected organ-system comorbidity categories recorded at admission (range 0–9).

### 2.4. Outcome

The primary outcome was 30-day all-cause mortality, ascertained through the hospital electronic record and the regional civil registry. Patients discharged alive before day 30 were considered survivors at 30 days only when survival was confirmed through follow-up records; otherwise, in keeping with conservative analytic practice, the date of the last known contact was used as the censoring point. No patient was lost to follow-up before day 7.

### 2.5. Statistical Analysis

Continuous variables are summarized as median [interquartile range, IQR] given the non-normal distribution of the inflammatory indices verified by the Shapiro–Wilk test. Categorical variables are reported as absolute count (percentage). Between-group comparisons used the Mann–Whitney U-test for continuous variables and Fisher’s exact test or the χ^2^ test for categorical variables, as appropriate. Inflammatory indices were natural-log-transformed prior to inclusion in regression models because of right-skewness; standardized log-transformed values are reported per 1 standard deviation increase to permit direct cross-marker comparison.

Discrimination was quantified using the area under the receiver operating characteristic curve (AUROC) with 95% confidence intervals derived from 2000 stratified bootstrap resamples. Pairwise comparisons of AUROC used the DeLong test as implemented from the Sun & Xu (2014) covariance formulation [17]. Optimal cut-offs were derived using the Youden index, with corresponding sensitivity, specificity, positive predictive value (PPV) and negative predictive value (NPV) at the relevant population prevalence.

To assess independent prognostic association, we fitted multivariable logistic regression models including each candidate index together with APACHE II and age, both standardized. The choice of a parsimonious adjustment set (three predictors) reflects the available 33 events and the canonical events-per-variable rule of approximately ten [18]; we verified through bootstrap that adding additional covariates produced unstable estimates without improving model fit. Mechanical ventilation was not included as a covariate in the primary adjusted models because it was considered a priori to lie predominantly on the causal pathway between sepsis severity and death (i.e., a mediator rather than a confounder), so that conditioning on it could bias the estimated index–mortality association. We acknowledge that this causal interpretation is an assumption rather than a demonstrated structure; we therefore did not rely on it, and instead verified in a sensitivity analysis (Section 3.9) that the AISI–mortality association persisted when mechanical ventilation was explicitly entered as a covariate. The dose–response relationship between each index and mortality was visualized using locally weighted regression (LOWESS, fraction = 0.6).

All 127 patients in the primary analytic cohort had complete admission complete blood count data and 30-day vital status; no imputation was required.

Incremental value over APACHE II was assessed using two complementary metrics. The integrated discrimination improvement (IDI) quantifies the change in mean predicted probability between events and non-events, and the continuous net reclassification improvement (cNRI) quantifies the proportion of correctly reclassified subjects, both following the Pencina et al. formulation [19]. We additionally report the Hosmer–Lemeshow goodness-of-fit statistic with ten deciles of risk and the Brier score for each model.

Clinical utility was further assessed using decision curve analysis, which plots net benefit (true positives minus weighted false positives) of competing prediction strategies across a range of threshold probabilities. We compared APACHE II + AISI, APACHE II alone, APACHE II + SII, and the reference “treat all” and “treat none” strategies across threshold probabilities from 5% to 70%, focusing on the 20–50% range as clinically relevant for ICU mortality triage decisions. Net benefit differences greater than 0.01 within this range were considered clinically meaningful.

Internal validation used 1000 bootstrap replications with apparent and test-sample AUROCs computed for each replicate; the mean optimism (apparent − test) was subtracted from the apparent AUROC to yield the optimism-corrected estimate, following Harrell [20]. All analyses were performed in Python (version 3.11) using statsmodels (0.14), scikit-learn (1.4) and SciPy (1.12). Two-sided *p*-values < 0.05 were considered statistically significant; we did not apply formal correction for multiple comparisons as the primary analysis, given the exploratory nature of the head-to-head AUROC matrix and the strong intercorrelation among the candidate indices (which violates the independence assumption of the Bonferroni method). We did, however, additionally assess the effect of correcting across the family of pairwise DeLong comparisons, using both Benjamini–Hochberg control of the false discovery rate and the more conservative Holm–Bonferroni procedure; these sensitivity analyses are reported in the Results. 

During the preparation of this manuscript, the authors used an artificial-intelligence assistant (Claude, Anthropic, Claude Opus 4.7) solely for language refinement, grammar checking, and stylistic editing of the English text. No content, data analysis, or interpretation was generated by the tool. The authors have reviewed and edited the output and take full responsibility for the content of this publication.

## 3. Results

### 3.1. Cohort Characteristics

Of 169 consecutive ICU sepsis admissions screened, 127 patients aged ≥65 years constituted the primary analytic cohort. Median age was 73 [IQR 69–79] years; 50.4% were male. The most common comorbidities were cardiovascular disease (56.7%), hypertension (46.5%) and neurologic illness (30.7%). Median admission SOFA was 9 [7–10] and APACHE II 28 [26–30], confirming severe illness. Thirty-day mortality was 26.0% (33/127), comparable to contemporary European ICU registries for elderly sepsis [3,5]. As expected, non-survivors were older (77 [73–79] vs. 72 [68–79] years, *p* = 0.024), more often male (72.7% vs. 42.6%, *p* = 0.005), more frequently on mechanical ventilation (81.8% vs. 9.6%, *p* < 0.001), and had higher admission APACHE II (30 [29–31] vs. 27 [24–29], *p* < 0.001). Comorbid pulmonary disease, malignancy, renal disease and clinical hepato-gastrointestinal disorders were over-represented among non-survivors. Detailed baseline data are presented in Table 1.

### 3.2. Distribution of Inflammatory Indices

**Figure 1:**
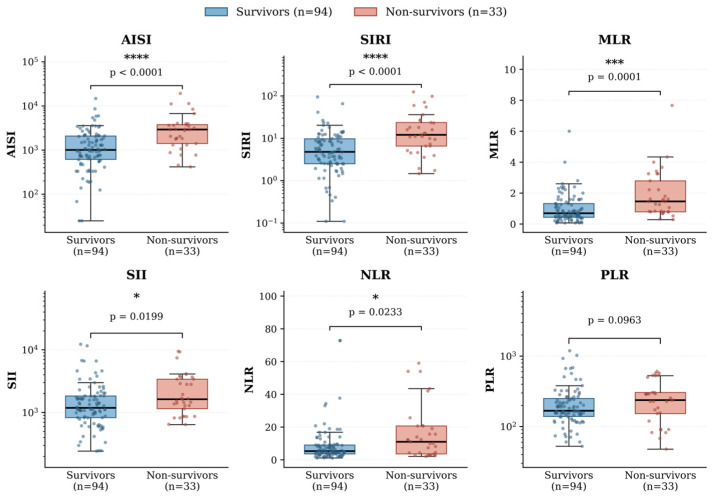
Distribution of hematological inflammatory indices by 30-day survival status, elderly sepsis cohort (*n* = 127). Box plots display median, interquartile range and 1.5 × IQR whiskers; outliers are shown as individual points; jittered overlay shows individual values. The y-axis is logarithmic for AISI, SIRI, SII and PLR. Mann–Whitney *p*-values are displayed in panel titles. The order of effect size, AISI > SIRI ≈ MLR > SII > NLR > PLR, is identical to the discrimination order shown in Figure 2. Significance levels are indicated as follows: * *p* < 0.05, *** *p* < 0.001, **** *p* < 0.0001.

All six hematological indices were higher among non-survivors than survivors at the unadjusted level, but the magnitude and statistical confidence of the difference varied substantially across markers (Figure 1). Median AISI was approximately threefold higher in non-survivors than survivors (2914 vs. 1009, *p* < 0.001) and median SIRI was 2.5-fold higher (12.07 vs. 4.79, *p* < 0.001). MLR demonstrated the largest relative difference between groups (1.46 vs. 0.69, *p* < 0.001). By contrast, the median between-group difference in SII (1623 vs. 1188, *p* = 0.020) was smaller, and the corresponding difference in PLR did not reach significance (*p* = 0.096). Notably, the absolute monocyte count was significantly higher in non-survivors (1.30 vs. 0.96 × 10^9^/L, *p* = 0.003), whereas absolute lymphocyte and platelet counts did not differ. This pattern is mechanistically informative: the differential in non-survivors is dominated by a monocyte-and-neutrophil signal rather than by lymphocyte depletion or thrombocytopenia, and indices that include the monocyte count therefore amplify the relevant prognostic signal.

### 3.3. Discriminative Performance for 30-Day Mortality

**Figure 2:**
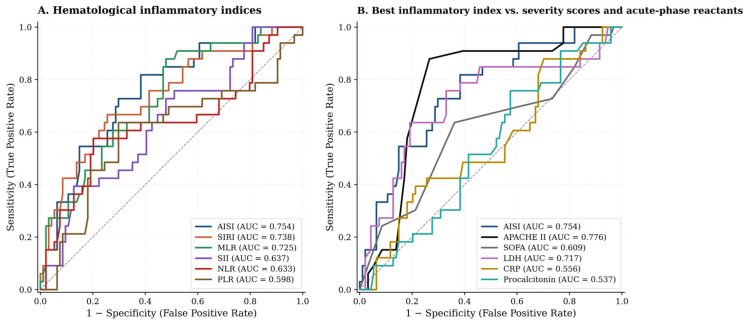
Receiver operating characteristic curves for 30-day mortality. (**A**) Comparison of the six hematological inflammatory indices in the elderly sepsis cohort. AISI, SIRI and MLR cluster at AUROC ≈ 0.73–0.75, with the curves separating from those of SII, NLR and PLR predominantly at the high-specificity (left) portion of the curve. (**B**) Comparison of AISI with the severity scores APACHE II and SOFA, and with the conventional acute-phase reactants C-reactive protein and procalcitonin, in the same cohort. AISI matches APACHE II in discriminative performance and clearly exceeds CRP, PCT and SOFA.

**Figure 3:**
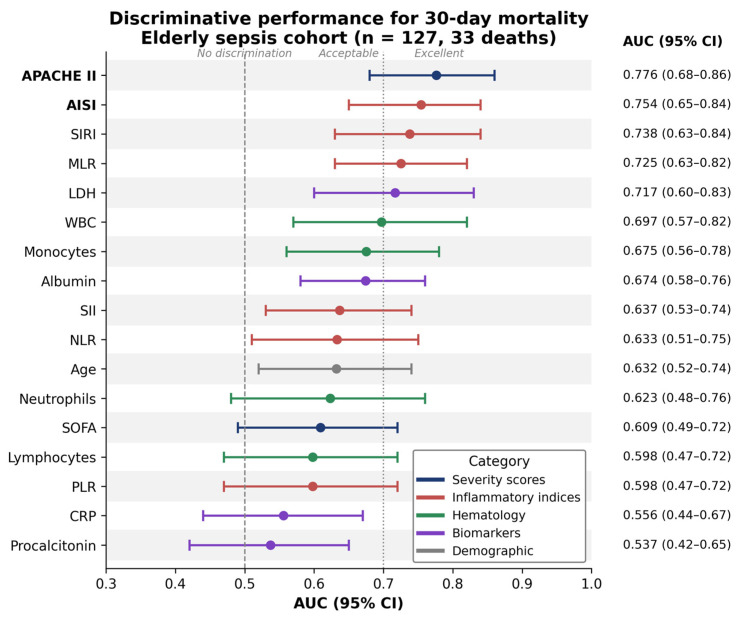
Forest plot of AUROC with 95% bootstrap confidence intervals for all candidate predictors examined in the elderly sepsis cohort. The dashed vertical line at 0.5 marks no discrimination; the dotted line at 0.7 marks the conventional threshold for clinically useful discrimination. Three distinct tiers are evident: severity-score and monocyte-containing indices (AISI, SIRI, MLR, APACHE II); intermediate markers including LDH, WBC, monocytes and albumin; and indices not reaching the 0.7 threshold (SII, NLR, age, SOFA, PLR, CRP, PCT).

AUROC values with 95% bootstrap confidence intervals are summarized in Table 2 and visualized in Figure 2 and Figure 3. Among inflammatory indices, AISI showed the highest discrimination (AUROC 0.754, 95% CI 0.654–0.845), followed by SIRI (0.738, 0.632–0.840), MLR (0.725, 0.626–0.824), SII (0.637, 0.526–0.744), NLR (0.633, 0.513–0.751) and PLR (0.598, 0.468–0.723). For comparison, APACHE II reached an AUROC of 0.776 (0.679–0.856), only marginally above AISI, whereas SOFA had limited discrimination (0.609, 0.490–0.720). C-reactive protein and procalcitonin performed at chance level (0.556 and 0.537, respectively), an unsurprising finding given that both are diagnostic rather than prognostic markers in sepsis [21]. LDH (0.717) was an unexpectedly competitive single marker.

Pairwise DeLong tests showed that AISI was significantly superior to SII (ΔAUROC +0.117, *p* = 0.009), NLR (+0.121, *p* = 0.032), PLR (+0.156, *p* = 0.046), CRP (+0.197, *p* = 0.005) and PCT (+0.217, *p* = 0.004); the difference between AISI and APACHE II was not significant (ΔAUROC −0.023, *p* = 0.74), nor was the difference between AISI and SIRI or MLR (Table 3). SIRI was significantly superior to SII (*p* = 0.024), NLR (*p* = 0.010), CRP and PCT. By contrast, the comparisons between SII, NLR, PLR and the conventional acute-phase reactants did not reach significance, indicating that these indices occupied the same lower discriminative tier. To gauge robustness to multiple-comparison adjustment, we additionally corrected the family of pairwise DeLong comparisons. Under Benjamini–Hochberg control of the false discovery rate, all comparisons significant at the nominal level remained significant (q ≤ 0.046), including the primary AISI-versus-SII contrast (q = 0.020). Under the more conservative Holm–Bonferroni procedure, the AISI-versus-CRP and AISI-versus-PCT comparisons remained significant, whereas the AISI-versus-SII comparison became marginal (Holm-adjusted threshold 0.0083 versus an observed *p* = 0.009). We interpret the central finding as robust to false-discovery control but acknowledge that it does not survive the most stringent family-wise correction. Spearman correlations between the indices (Supplementary Table S3 and Supplementary Figure S1) confirmed that AISI, SIRI and MLR carry substantially overlapping information (ρ ≥ 0.81 among these three), while APACHE II showed near-zero correlation with all inflammatory indices (|ρ| ≤ 0.14), supporting the mechanistic complementarity that underlies the combined APACHE II + AISI model. Because AISI, SIRI and MLR are mathematically related and strongly intercorrelated, we do not regard AISI as biologically distinct from the other two; rather, we use it throughout as a single representative exemplar of the monocyte-containing class (it had the numerically highest point AUROC of the three, 0.754, although it was not statistically superior to SIRI or MLR), and SIRI or MLR could be substituted in the combined model with essentially equivalent results. Our central claim is thus a claim about the monocyte-containing class relative to SII and NLR, not a claim that AISI uniquely captures prognostic biology.

### 3.4. Independent Association with Mortality

In univariable logistic regression, the standardized log-AISI yielded the largest odds ratio (OR per 1 SD = 3.05, 95% CI 1.73–5.38, *p* < 0.001), followed by log-SIRI (2.82, 1.65–4.84, *p* < 0.001) and log-MLR (2.71, 1.60–4.62, *p* < 0.001). Log-SII (1.68, 1.11–2.54, *p* = 0.014), log-NLR (1.71, 1.14–2.56, *p* = 0.010) and log-PLR (1.25, 0.85–1.86, *p* = 0.260) were either weakly or non-significantly associated with mortality. After adjustment for APACHE II and age (Table 4), the effect sizes attenuated for all markers, but the contrast remained pronounced. Log-AISI retained an adjusted OR of 2.80 (1.54–5.08, *p* = 0.001), and APACHE II remained independently associated (aOR per 1 SD = 2.37, *p* = 0.004). In the corresponding model containing log-SII, the marker became non-significant (aOR 1.54, 95% CI 0.99–2.39, *p* = 0.058) while APACHE II remained the dominant predictor; log-PLR was clearly null after adjustment (aOR 1.17, *p* = 0.482).

**Figure 4:**
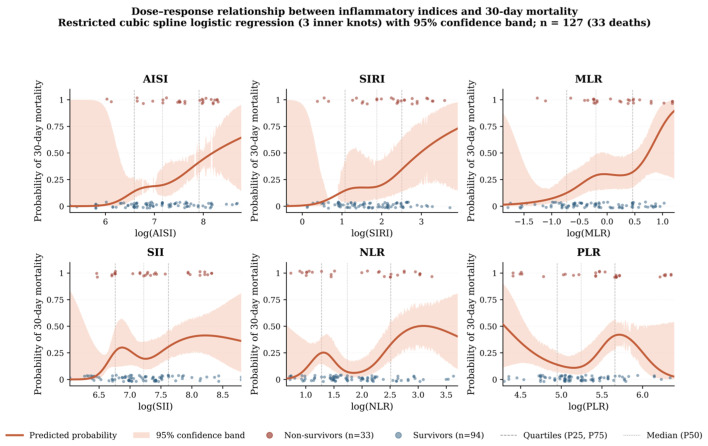
LOWESS-smoothed dose–response curves between log-transformed inflammatory indices and 30-day mortality (probability scale on y-axis). Dotted vertical lines mark the 25th, 50th and 75th percentiles of each marker. AISI, SIRI and MLR show steep monotonic ascending relationships; SII and NLR are essentially flat across most of the observed range; PLR shows a non-monotonic pattern that probably reflects the unstable behavior of the platelet count in advanced sepsis.

The dose–response relationships visualized in Figure 4 corroborate this finding. AISI, SIRI and MLR show steep, monotonic ascending curves with a near-saturation behavior at high values. SII and NLR, in contrast, are essentially flat across most of their range: extreme values are present in non-survivors, but the bulk of the distribution carries little mortality discrimination. PLR shows a non-monotonic pattern. The flat LOWESS shape for SII and NLR is mechanistically interpretable: these indices are dominated by the platelet count (in the case of SII) and by the lymphocyte denominator (NLR), both of which can be elevated for non-prognostic reasons in elderly inflamed patients.

### 3.5. Incremental Value over APACHE II

**Figure 5:**
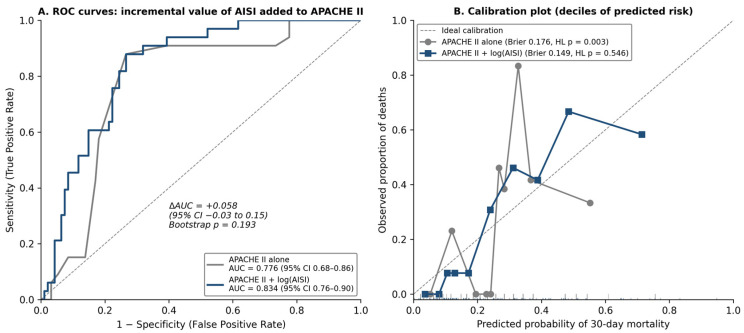
(**A**) ROC curves for APACHE II alone and the APACHE II + AISI combined logistic model. The curves overlap at the high-sensitivity end but the combined model is uniformly closer to the upper-left corner across the clinically relevant high-specificity region. (**B**) Decile-based calibration plot. The combined APACHE II + AISI model shows close adherence to the diagonal line of perfect calibration; APACHE II alone exhibits visible deviations particularly in the highest decile, where it under-predicts observed mortality.

Although the difference in AUROC between AISI and APACHE II was not statistically significant when each was used in isolation, the question of greater clinical interest is whether adding AISI to APACHE II improves prediction. The combined APACHE II + AISI logistic model achieved an AUROC of 0.834 in the apparent dataset, compared to 0.776 for APACHE II alone (ΔAUROC +0.058, DeLong *p* = 0.20; Figure 5A). On our primary, most conservative discrimination test, therefore, the addition of AISI did not significantly improve the AUROC, and we do not claim a discrimination benefit on this basis. The reclassification metrics, which are more sensitive but are also known to be susceptible to inflation in small samples and do not by themselves establish clinical usefulness, are reported as supportive rather than confirmatory evidence: adding AISI to APACHE II improved the IDI by 0.139 (*p* < 0.001), meaning that the average predicted probability for non-survivors increased by 13.9 percentage points more than the corresponding increase among survivors. The continuous NRI was 0.671 (*p* < 0.001), with both events (+0.394) and non-events (+0.277) contributing positively. SIRI and MLR yielded similar gains (IDI 0.134 and 0.120 respectively, both *p* < 0.001; cNRI 0.567 and 0.571, both *p* < 0.01). In contrast, SII added a marginal IDI of 0.039 (*p* = 0.025) and a non-significant cNRI of 0.137 (*p* = 0.50). NLR yielded an intermediate cNRI (0.507, *p* = 0.012) but a low IDI of 0.051 (Table 5).

Calibration was acceptable for all combined models. The Hosmer-Lemeshow χ^2^ for APACHE II + AISI was 9.94 (8 df, *p* = 0.27), with a calibration plot showing close agreement between predicted and observed mortality across deciles of risk (Figure 5B). The Brier score decreased from 0.176 for APACHE II alone to 0.148 for APACHE II + AISI, a relative reduction of 16%. When APACHE II was modeled as the sole predictor of 30-day mortality in a univariate logistic regression in our cohort, calibration was suboptimal (HL χ^2^ = 40.4, *p* < 0.001), with under-prediction of risk in the lowest deciles. This likely reflects case-mix differences from the cohorts in which APACHE II was originally derived rather than an intrinsic property of the score.

### 3.6. Internal Validation

Bootstrap optimism correction confirmed the robustness of the combined APACHE II + AISI model. Across 1000 bootstrap replications, the mean optimism was 0.010, yielding an optimism-corrected AUROC of 0.824 (compared with the apparent 0.834). For APACHE II alone, the corresponding optimism was negligible (−0.001), and the optimism-corrected AUROC was essentially identical to the apparent value (0.778). The very small optimism for the combined model (an order of magnitude lower than typically reported in clinical risk-prediction studies of comparable size [22]) is consistent with the parsimonious model specification (two predictors) and the use of standardized predictors which limits coefficient instability.

### 3.7. Decision Curve Analysis

**Figure 6:**
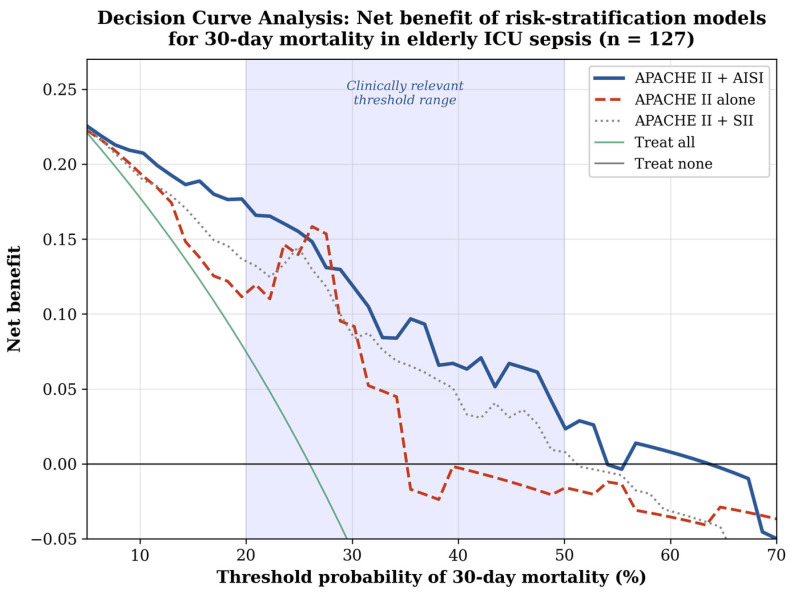
Decision curve analysis for 30-day mortality prediction in elderly ICU sepsis (*n* = 127). Net benefit is plotted against threshold probability of mortality for: APACHE II + AISI (solid blue), APACHE II alone (dashed red), APACHE II + SII (dotted gray), and the “treat all” (green) and “treat none” (black, x-axis) reference strategies. The shaded region (20–50%) indicates the clinically relevant range of threshold probabilities for ICU decision-making. APACHE II + AISI delivers higher net benefit than all alternatives across this range and remains positive beyond the threshold at which APACHE II alone becomes inferior to “treat none”.

As a secondary, model-based analysis of potential clinical utility, we performed decision curve analysis across threshold probabilities from 5% to 70% (Figure 6). Across the clinically relevant range of threshold probabilities for ICU mortality (20–50%), the combined APACHE II + AISI model demonstrated higher net benefit than either APACHE II alone, “treat all” or “treat none” strategies. At a threshold of 20%, net benefit was 0.177 for APACHE II + AISI versus 0.112 for APACHE II alone (ΔNB +0.065; under the assumptions of decision curve analysis this corresponds to a net of approximately 6.5 additional true high-risk patients identified per 100 at the same false-positive rate, a model-based quantity that does not itself represent a measured change in patient outcome; Supplementary Table S6). At thresholds ≥35%, APACHE II alone yielded negative net benefit relative to the “treat none” reference, while APACHE II + AISI retained positive net benefit up to a threshold of 55%. The APACHE II + SII model performed intermediately and consistently below APACHE II + AISI across the entire range, confirming the analytical hierarchy observed in the discrimination and reclassification analyses.

### 3.8. Two-Step Risk Stratification Pathway

**Figure 7:**
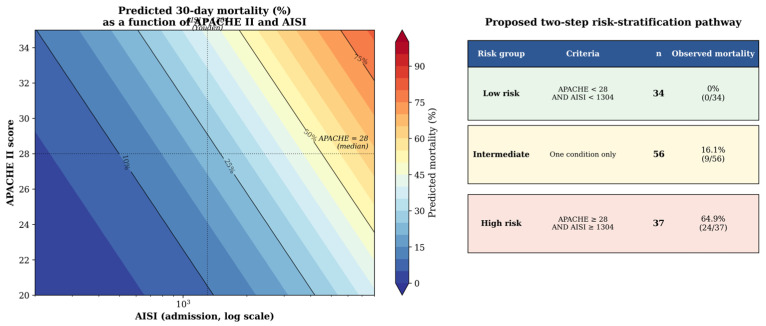
Combined APACHE II + AISI prediction model and proposed two-step risk-stratification pathway. (**Left**) Risk surface showing predicted 30-day mortality (%) for each combination of APACHE II score (y-axis) and admission AISI (x-axis, logarithmic). Contour lines denote 10%, 25%, 50% and 75% risk. Dotted lines mark the APACHE II median (28) and the Youden-optimal AISI cut-off (1304). The underlying model is a two-predictor logistic regression: LP = −11.98 + 0.128 × APACHE II + 0.997 × ln(AISI). (**Right**) Cross-classification of the elderly cohort (*n* = 127) by these two thresholds yields three risk strata with markedly distinct 30-day mortality (0% in low-risk, 16.1% intermediate, 64.9% in high-risk).

As an exploratory illustration of how the combined APACHE II + AISI model might inform risk stratification, we constructed a two-step pathway using the median APACHE II (28) and the Youden-optimal AISI cut-off (1304) as cut-points. The cohort was partitioned into three risk groups (Figure 7). The low-risk group (APACHE II < 28 AND AISI < 1304, *n* = 34, 26.8% of cohort) experienced zero deaths at 30 days (observed mortality 0%, 0/34). The high-risk group (APACHE II ≥ 28 AND AISI ≥ 1304, *n* = 37, 29.1% of cohort) had an observed mortality of 64.9% (24/37). The intermediate group (one but not both criteria met, *n* = 56) had a mortality of 16.1% (9/56). The full multivariable logistic model (predicted mortality = exp(LP)/(1 + exp(LP)), where LP = −11.98 + 0.128 × APACHE II + 0.997 × ln(AISI)) is presented graphically in Figure 7 (left panel) as a risk surface, providing patient-level probability estimates from any combination of admission APACHE II and AISI values.

### 3.9. Sensitivity Analyses

The robustness of the AISI signal was explored across multiple secondary sensitivity analyses. Adjustment for sex (in addition to APACHE II and age) preserved the AISI effect (aOR per 1 SD = 2.68, 95% CI 1.49–4.80, *p* = 0.001) and similarly preserved SIRI (aOR 2.57, 1.47–4.51, *p* = 0.001) and MLR (aOR 2.43, 1.39–4.26, *p* = 0.002), while SII remained non-significant (aOR 1.54, 0.98–2.43, *p* = 0.061). To address the possibility of residual confounding by the observed baseline imbalances, the AISI association was additionally re-examined in models that added, individually, mechanical ventilation (aOR per 1 SD 2.61, 95% CI 1.13–6.03, *p* = 0.025) and the total number of comorbidities (aOR 2.14, 1.09–4.21, *p* = 0.028); in each case AISI remained independently associated with mortality. In a fully saturated model simultaneously including sex, mechanical ventilation and comorbidity count (six predictors for 33 events, well beyond the events-per-variable limit), the AISI effect retained a comparable magnitude (aOR 2.57) but reached only marginal significance (95% CI 0.98–6.72, *p* = 0.054), an attenuation attributable to over-parameterization rather than to loss of signal, and one that underscores why the pre-specified parsimonious model was the appropriate primary specification. Adjustment for LDH (the strongest non-inflammatory continuous predictor available) attenuated but did not abolish the AISI effect (aOR 2.38, 1.28–4.42, *p* = 0.006). Exclusion of patients with active non-hematological malignancy (remaining *n* = 93, 15 deaths) preserved the AISI signal (AUROC 0.769, 95% CI 0.635–0.888; adjusted aOR 2.54, 1.13–5.70, *p* = 0.024), excluding the possibility that the AISI association is driven by malignancy-related leukocytosis. Descriptive analysis stratified by source of infection (Supplementary Table S4) showed consistently higher median AISI in non-survivors than survivors across respiratory, urogenital and gastrointestinal sources, supporting the source-independent nature of the AISI–mortality association, although subgroup sizes precluded inferential testing. Subgroup analysis by mechanical ventilation status, a proxy for severity of organ dysfunction, showed that AISI performed strongly in both subgroups (AUROC 0.835 in ventilated patients, 0.706 in non-ventilated; Supplementary Figure S2), with the largest separation from SII and NLR observed in the ventilated subgroup, where AISI exceeded APACHE II in discrimination (0.835 vs. 0.704). Detailed sensitivity results are summarized in Supplementary Table S1. The complete list of alternative AISI cut-offs and their operational characteristics is provided in Supplementary Table S2.

### 3.10. Performance in Patients Younger than 65

In the exploratory descriptive comparison with the small group of patients aged <65 years (*n* = 42, 1 death), formal AUROC analysis was uninformative because of the single event. We note descriptively that the inflammatory indices were of comparable magnitude in this group (median AISI 678 [261–2510]; median SII 1548 [1000–3902]). The single non-survivor in the <65 group had AISI of 3176 (above the cohort 75th percentile for AISI of 2732) and SII of 1176 (well below the cohort 75th percentile for SII of 2045), but no inferential conclusion can be drawn from a single event.

## 4. Discussion

In this single-center cohort of 127 elderly patients admitted to a tertiary ICU with Sepsis-3-defined sepsis, hematological inflammatory indices that explicitly incorporate the absolute monocyte count (AISI, SIRI and MLR) were stronger predictors of 30-day mortality than the more widely studied SII and NLR, with the difference reaching statistical significance for AISI versus SII and NLR specifically. AISI matched APACHE II in stand-alone discrimination (AUROC 0.754 vs. 0.776) and improved both reclassification and calibration when added to it. The principal contribution of this report is therefore to question, rather than to overturn, the prevailing hierarchy of hematological prognostic markers in sepsis as it has been established in mixed-age cohorts, particularly the influential study by Ou et al. in 469 patients with bloodstream infection [10], in which SII and NLR were declared the optimal markers.

### 4.1. Why the Elderly Differ

The discordance between our findings and the published literature is best understood through the lens of immunosenescence and inflammaging. Aging alters baseline circulating leukocyte composition in directions that specifically degrade the prognostic specificity of NLR and the SII derivative. The naïve T-cell pool contracts after thymic involution, and a substantial fraction of circulating CD8^+^ T cells in older adults is of the terminally differentiated, often clonally restricted CD45RA^+^CCR7^−^ phenotype [11,23]. Resting-state lymphocyte counts in healthy adults over 75 are typically 10–20% lower than in adults aged 30–50, and the resting NLR rises with age even in the absence of acute infection [24,25]. By implication, an NLR of, say, nine (well above conventional cut-offs proposed in earlier sepsis cohorts) may represent a much smaller deviation from baseline in an octogenarian than in a middle-aged adult. The diagnostic threshold therefore loses specificity, and the associated AUROC degrades.

Platelets have a parallel issue. In late sepsis, platelet count behavior is bidirectional: thrombocytosis is common in early bacterial infection, particularly with Streptococcus pneumoniae or Klebsiella, whereas septic disseminated intravascular coagulation (DIC) and bone-marrow suppression produce thrombocytopenia later [26]. The platelet count therefore does not co-vary monotonically with severity, and any index dominated by it (SII, PLR) inherits this ambiguity. The flat LOWESS curves for SII and NLR in our cohort (Figure 5) are the empirical signature of this poor monotonicity.

Monocytes, by contrast, become more informative with advancing age in the context of sepsis. Aging is associated with expansion of the intermediate (CD14^++^CD16^+^) and non-classical (CD14^+^CD16^++^) monocyte subsets, which together are the principal cellular sources of TNF-α, IL-6 and IL-1β in early sepsis [13,27]. Monocyte distribution width (MDW), a flow-cytometric quantification of cell-size heterogeneity, was approved by the U.S. FDA in 2019 as a stand-alone biomarker for sepsis triage in emergency departments precisely because of this association [14,28]. In our cohort the absolute monocyte count was significantly higher in non-survivors (1.30 vs. 0.96 ×10^9^/L, *p* = 0.003), even though the absolute lymphocyte count and platelet count did not differ between groups. One interpretation consistent with this pattern is that the monocyte compartment carries prognostic information that the indices incorporating monocytes (AISI, SIRI, MLR) capture, whereas this remains an inference from the cell-count distribution rather than a directly demonstrated mechanism.

We emphasize that this account is a biologically plausible interpretation rather than a demonstrated causal mechanism. Our data are derived entirely from the differential complete blood count; we did not measure monocyte HLA-DR, monocyte distribution width, cytokines, or any functional immune marker. The proposed monocyte-driven explanation should therefore be regarded as a hypothesis generated by the observed association pattern, to be tested directly in studies incorporating mechanistic immunophenotyping, rather than as a conclusion established by the present analysis.

### 4.2. Comparison with Published Cohorts

Our results contrast most directly with Ou and colleagues, who reported in their bloodstream-infection cohort that SII (AUROC 0.760) and NLR (0.765) were the leading predictors and that SIRI (0.719), AISI (0.701), MLR (0.745) and PLR (0.740) trailed them [10]. Two factors plausibly account for the divergence. First, their cohort had a median age below 65 and explicitly excluded patients on immunosuppressants and chemotherapy, but mixed inpatients and presumably less severely ill cases (the proportion requiring mechanical ventilation was not reported). The age-driven loss of NLR specificity discussed above would not have manifested in their population. Second, their analysis adjusted only for demographics and comorbid disease, not for severity of illness scores, so unconfounded effects of inflammation on survival could not be separated from confounding by overall acuity. Our adjustment for APACHE II (Table 4) eliminated the SII and PLR signals while preserving those of AISI, SIRI and MLR, which suggests that part of the previously reported NLR/SII effect was attributable to severity that the indices indirectly capture.

In the broader literature, our findings are consistent with several recent reports. A meta-analysis of 12 studies and 10,811 adult sepsis patients by Huang et al. [29] showed that high admission NLR is associated with poor prognosis (pooled hazard ratio approximately 1.75), but with substantial between-study heterogeneity and inconsistent cut-off values across cohorts, supporting the view that NLR carries useful but limited prognostic information. In a Polish ICU cohort of septic shock patients, Liberski et al. [30] evaluated NLR, PLR, MLR and the platelet/MPV ratio and concluded that, while NLR helped identify multi-organ failure, none of the haemogram-derived indices was associated with mortality in that setting, further illustrating that the prognostic performance of these markers depends heavily on the specific population studied. In very-low-birth-weight neonates with early-onset sepsis, Cakir & Tayman [31] compared six systemic inflammatory indices (NLR, PLR, MLR, SII, PIV and SIRI) and identified SIRI as the most useful, again indicating that monocyte-containing indices may outperform the platelet-driven SII outside the typical mid-life adult cohort. The relevant counter-evidence is the recent MIMIC-IV analysis by Pan et al. in 976 patients with acute respiratory distress syndrome [32], in which a higher SII (≥1694) remained an independent predictor of 30-day mortality even after adjustment for SAPS II and APS III (HR 1.38, 95% CI 1.08–1.77, *p* = 0.002). Notably, Pan et al. did not include monocyte-based indices in their analysis, so a direct comparison with AISI or SIRI in their cohort is not available; their finding does not contradict ours, but it underscores that SII may retain prognostic value in some critically ill populations even when, as in our cohort, it is outperformed by monocyte-based alternatives in elderly sepsis.

The population-dependence of hematological index performance is well illustrated by the chronic heart failure (CHF) literature, where systemic inflammation reflects a sustained, comorbidity-driven process rather than the acute immunosenescent dysregulation that characterizes elderly sepsis. In contrast to our findings, the platelet- and neutrophil-driven SII has shown robust prognostic value across the CHF spectrum: in a cohort of 2748 CHF patients, elevated SII was independently associated with all-cause mortality across all ejection–fraction categories (with an optimal cut-off near 917 and a 44% increase in mortality risk in the high-SII group), and in heart failure with preserved ejection fraction SII outperformed SIRI, NLR and CRP for cardiovascular mortality and rehospitalization. This apparent contradiction with our results is, in fact, mechanistically coherent: in CHF the prognostic signal is dominated by chronic neutrophil- and platelet-mediated low-grade inflammation and cardiac remodeling, for which SII is well suited, whereas in acute elderly sepsis the monocyte compartment carries the dominant prognostic information. Rather than undermining either body of work, this contrast reinforces our central thesis that the optimal hematological index is contingent on the underlying disease biology and the age structure of the population, and that index thresholds should not be transported uncritically between clinical contexts [33,34].

Beyond the acute critical-care setting, both SII and SIRI have been extensively studied as predictors of long-term all-cause and cardiovascular mortality in chronic metabolic and inflammatory conditions. In a 20-year follow-up cohort of 42,875 US adults, higher SII and SIRI were independently associated with increased all-cause and cardiovascular mortality, and analogous associations have been reported across metabolically defined populations: in 13,026 obese adults from NHANES, SIRI predicted both all-cause and cardiovascular death and outperformed SII, and elevated SIRI has likewise been linked to cardiovascular and all-cause mortality in metabolic dysfunction-associated steatotic liver disease and diabetic nephropathy. Beyond leukocyte ratios, related composite indices integrating nutritional and atherogenic status, such as the prognostic nutritional index and the atherogenic coefficient, have also been associated with obesity and metabolic indices in diabetic nephropathy [35], underscoring that complete-blood-count- and metabolism-derived markers jointly track risk across the spectrum of chronic inflammatory-metabolic disease. A consistent theme across these chronic-inflammatory cohorts is that the monocyte-containing SIRI tends to track cardiovascular and all-cause mortality at least as strongly as the platelet-driven SII, paralleling, in a very different clinical context, the superiority of monocyte-containing indices that we observed in acute elderly sepsis. While metabolic inflammatory syndrome and ICU sepsis are mechanistically distinct, this convergence suggests that the monocyte compartment may carry prognostic information that the platelet–neutrophil axis of the SII does not fully capture whether the inflammatory stimulus is chronic and metabolic or acute and infectious [36,37].

A direct comparison among SIRI, NLR and MLR within our cohort addresses this question empirically. Both monocyte-containing indices clearly outperformed NLR: SIRI (AUROC 0.738) and MLR (AUROC 0.725) discriminated 30-day mortality substantially better than NLR (AUROC 0.633), and after adjustment for APACHE II and age both SIRI (adjusted OR per 1 SD 2.65) and MLR (2.64) remained independently associated with mortality, whereas NLR did not. Between SIRI and MLR, however, performance was virtually identical and the two indices were strongly correlated (ρ ≥ 0.81), so our data do not support a meaningful prognostic distinction between them; both should be regarded as capturing the same monocyte-driven signal. The practical implication is that NLR, despite being the most widely reported index in the sepsis literature, is the weakest of the three in elderly ICU sepsis, while either SIRI or MLR can be used interchangeably as a simple, monocyte-informed alternative. This is consistent with reports outside the typical mid-life adult cohort, such as the identification of SIRI as the most useful index in very-low-birth-weight neonatal sepsis, and reinforces our broader thesis that monocyte-containing indices are more strongly associated with mortality than the neutrophil-driven NLR, while being near-equivalent and strongly intercorrelated among themselves.

### 4.3. Clinical Implications

The following clinical implications are offered as hypotheses for prospective evaluation rather than as recommendations for current practice; none follows directly from a retrospective, internally validated, single-center analysis, and each would require external and ideally clinical-impact validation before adoption.

If the index of choice in elderly sepsis is AISI rather than SII, three practical implications follow. First, the often-cited “breakpoint” of 1711 for SII reported by Ou et al. [10], which has been used as a triage cut-off in some clinical decision-support implementations, is poorly applicable in elderly ICU patients: in our cohort, SII at 1711 has sensitivity of only 42% and specificity of 70%, with a Youden index of 0.13. By contrast, AISI at the Youden-optimal cut-off of 1304 reaches an apparent sensitivity of 82% with NPV of 91% (optimism-corrected ≈77% and ≈89%; see Limitations), suggesting that, subject to external recalibration, it could be explored as a high-sensitivity safety-net at ICU triage. Second, the addition of AISI to APACHE II, which adds no incremental cost since both are calculated from data already available within the first 24 h, produces a measurable improvement in calibration and reclassification (cNRI 0.67), within the range that has been considered clinically meaningful in cardiovascular risk prediction [19,38]. Third, because the differential signal in non-survivors in our data resides in monocytes rather than lymphocytes or platelets, our findings provide indirect support for current efforts to integrate monocyte-specific markers (MDW, monocyte HLA-DR) into sepsis risk stratification protocols [14]. AISI could be investigated as a low-cost proxy for these more sophisticated assays in centers without flow cytometry capability, although this potential role is hypothetical and would require dedicated comparative and prospective study.

The decision curve analysis (Figure 6) quantifies the clinical translation of these findings. Across the clinically meaningful range of threshold probabilities for ICU mortality (20–50%), APACHE II + AISI shows higher model-based net benefit than APACHE II alone. We note that the corresponding figure of 5–7 additional true high-risk patients per 100 is a theoretical quantity derived from the net-benefit calculation under fixed threshold assumptions; it reflects classification performance, not a demonstrated improvement in clinical outcomes, which would require prospective evaluation linking the strategy to interventions and patient-level endpoints. The proposed two-step pathway (Figure 7) is intended only to illustrate, descriptively, how the two markers co-vary with risk in our data: the high-risk stratum (APACHE II ≥ 28 AND AISI ≥ 1304) showed an observed 30-day mortality of 65%, while the low-risk stratum (both below threshold) had no deaths. We draw no clinical management recommendations from these strata. In particular, the apparent absence of deaths in the low-risk group must not be interpreted as supporting de-escalation of care, and the high-risk figure does not by itself justify any specific escalation; both observations are descriptive features of a single small cohort. The strata require prospective external validation before any clinical use, and the stark apparent contrast (0% vs. 65%) is in part an artifact of the modest cohort size and the small per-stratum counts (*n* = 34 and *n* = 37), and would be expected to attenuate substantially in larger samples.

Although our outcome was 30-day mortality rather than the progression to septic shock, the early prediction of haemodynamic deterioration is a closely related clinical priority, and the hematological indices examined here sit within a broader landscape of early sepsis biomarkers. Conventional markers, such as serum lactate, procalcitonin and presepsin, have been most extensively evaluated for the early identification of patients at risk of septic shock: in a prospective Sepsis-3 cohort, procalcitonin and presepsin were the best predictors of sepsis and septic shock (AUROC 0.711 and 0.709, respectively), outperforming CRP and lactate, while presepsin, PCT and APACHE II have repeatedly emerged as independent predictors of shock at emergency-department triage, alongside chronic kidney disease, malignancy, acute kidney injury and elevated LDH. Recent syntheses emphasize that the prognostic value of these biomarkers is strongly time-dependent: early inflammatory mediators such as IL-6 and TNF-α peak rapidly, whereas markers of endothelial injury, coagulation activation and immune suppression evolve more gradually, so that single time-point measurement may be insufficient and serial sampling at 6–24 h can improve risk stratification. This framework places our admission-based indices in context: AISI, SIRI and MLR are inexpensive, universally available single-time-point markers that may complement, rather than replace, lactate- and presepsin-based pathways for early shock prediction [39,40,41].

A further emerging biomarker that complements the leukocyte-derived indices examined here is serum butyrylcholinesterase (BChE), a liver-synthesized enzyme that hydrolyzes acetylcholine and serves as a peripheral index of the cholinergic anti-inflammatory pathway. In contrast to the inflammatory ratios, which rise with disease severity, BChE activity falls in systemic inflammation: reduced serum BChE has been shown to indicate severe systemic inflammation in critically ill patients and to correlate inversely with disease severity, and a sustained reduction in BChE activity measured at ICU admission discriminated 90-day survivors from non-survivors in sepsis, often earlier than IL-6 or TNF-α. Low admission cholinesterase activity has likewise been associated with 30-day mortality in Sepsis-3 cohorts and with severity and mortality in critically ill patients with COVID-19. Because BChE can be measured rapidly at the point of care, it has been proposed as a cost-effective adjunct for early risk stratification. Conceptually, BChE and the monocyte-containing indices probe complementary facets of the dysregulated host response, the cholinergic anti-inflammatory tone and the innate monocyte–neutrophil effector arm, respectively, and their combination represents an attractive, mechanistically grounded direction for future multimarker models in elderly sepsis [42,43,44].

### 4.4. Strengths and Limitations

This study has several strengths. The cohort is well-defined by Sepsis-3 criteria, the outcome is hard (30-day all-cause mortality) and the analytic approach is pre-specified. We use the appropriate severity scores (APACHE II, SOFA) as adjustment variables, an omission of the Ou et al. analysis, and we report multiple complementary metrics of incremental prognostic value (AUROC, IDI, cNRI, calibration, decision curve analysis), so that the conclusions do not rest on any single test. Robustness was further verified through pre-specified sensitivity analyses (adjustment for sex; adjustment for LDH; exclusion of malignancy patients; stratification by mechanical ventilation status), all of which preserved the AISI signal. Internal validation by bootstrap optimism correction was performed; the optimism was small. Comparison with alternative multi-marker model specifications (Supplementary Table S5) confirmed that the parsimonious APACHE II + AISI combination captures most of the achievable prognostic information; adding LDH yielded only a marginal AUROC increase (+0.011), and an APACHE-free alternative based on AISI + LDH + age (AUROC 0.778) approached APACHE II alone, an interesting finding for settings where APACHE II cannot be calculated routinely.

There are also material limitations. The single-center design and the modest sample size (*n* = 127, 33 events) place obvious bounds on external validity and on the precision of point estimates; the wide confidence intervals on the AUROCs (Table 2) are a direct reflection. In particular, the marginal non-significance of the DeLong test for the AISI-versus-APACHE II addition (*p* = 0.20) is in part a consequence of low power, and we have therefore been careful not to claim that AISI improves AUROC over APACHE II in the strict statistical sense; the IDI and cNRI are the more sensitive metrics that do reach significance. The retrospective design precludes inference about temporal dynamics: we measured indices only at admission, whereas dynamic monitoring may carry additional information [45]. The exclusion of immunosuppressed and onco-hematological patients limits applicability to those populations. The cohort was Romanian and predominantly Caucasian; ethnic differences in baseline leukocyte distributions, particularly the high prevalence of benign ethnic neutropenia in patients of African ancestry, may further modify these relationships [46]. The case-mix of sepsis in this region is also shaped by local microbiological epidemiology; the predominance of Gram-negative pathogens and their antimicrobial-resistance profiles in sepsis from the same south-western Romanian setting has been characterized previously [47], and such case-mix specificity further limits direct extrapolation of our findings to populations with different pathogen and resistance distributions. Finally, we did not measure monocyte HLA-DR, MDW, or cytokines, which would have permitted a direct mechanistic test of the monocyte-driven hypothesis; this is a logical next step. The Youden-optimal cut-off of 1304 for AISI was both derived and evaluated in the same sample; sensitivity, specificity and NPV at this threshold should therefore be interpreted as apparent values requiring prospective validation in independent cohorts. To quantify this optimism directly, we performed a bootstrap analysis (2000 resamples) in which the Youden-optimal cut-off was re-derived in each resample and evaluated on the original data. The optimism was modest but non-negligible: the apparent sensitivity of 82% and NPV of 91% corresponded to optimism-corrected values of approximately 77% and 89%, respectively. The cut-off itself was unstable across resamples (bootstrap median 1767, IQR 1304–1835), indicating that the specific value of 1304 should be regarded as an in-sample estimate requiring recalibration in external data rather than as a transportable threshold.

Finally, the ratio of analyses to events warrants caution: with 127 patients and 33 deaths, the cut-off-based operating characteristics and the risk strata in particular should be interpreted as exploratory and hypothesis-generating rather than as validated estimates, and the secondary, calibration, decision-curve and subgroup analyses are reported to characterize a single pre-specified model rather than as independently powered comparisons.

### 4.5. Future Directions

Three confirmatory lines of investigation are warranted. (i) Prospective multi-center validation of AISI in elderly ICU sepsis, ideally in a cohort large enough (≥500 patients with ≥100 deaths) to permit DeLong-significant AUROC comparisons against APACHE II. (ii) Integrated assessment of AISI alongside MDW and monocyte HLA-DR in a single cohort, to test whether these markers carry independent or redundant information. (iii) Trajectory analysis using serial blood counts in the first 72 h, the period during which sepsis bundle interventions are deployed, to determine whether AISI dynamics, rather than the admission value, are the more informative quantity.

Beyond conventional regression, machine-learning (ML) approaches are increasingly being applied to integrate hematological inflammatory indices into mortality-prediction models for geriatric sepsis. Recent work has shown that interpretable ML algorithms incorporating the SII can predict 28-day mortality in elderly sepsis with high apparent discrimination, an XGBoost model integrating admission SII in elderly patients with sepsis secondary to community-acquired pneumonia reached an AUROC of 0.901, with SII identified as a pivotal feature and a non-linear, U-shaped SII–mortality relationship, while other interpretable ML pipelines using SHAP and LASSO-based feature selection on large ICU databases have repeatedly retained inflammatory and CBC-derived variables (NLR, RDW, WBC, platelets) among their top predictors of 28-day mortality. These models offer flexibility in capturing non-linear effects and high-order interactions that a parsimonious logistic model cannot. We deliberately chose a transparent, low-dimensional logistic specification in the present study because our event count (33 deaths) does not support stable training of flexible ML models without substantial overfitting risk; nonetheless, the integration of monocyte-containing indices such as AISI and SIRI, which were not examined in these prior SII-focused ML studies, into interpretable, externally validated ML frameworks represents a promising avenue for future research in larger geriatric sepsis cohorts [48,49].

## 5. Conclusions

In a cohort of elderly ICU patients with Sepsis-3-defined sepsis, hematological inflammatory indices that incorporate monocytes (AISI, SIRI, MLR) were more strongly associated with 30-day mortality than the more widely studied platelet- and neutrophil-driven SII and NLR, both as stand-alone classifiers and when added to APACHE II; on pairwise testing this advantage reached statistical significance against SII and NLR specifically, whereas AISI, SIRI and MLR were strongly intercorrelated (ρ ≥ 0.81) and did not differ significantly from one another or from APACHE II. The optimism-corrected AUROC of the APACHE II + AISI combined model was 0.824, with concomitant improvements in reclassification (cNRI 0.67) and calibration. Within the limits of a single-center, retrospective cohort, these findings argue against the uncritical extrapolation of SII- and NLR-based risk thresholds to elderly patients and identify the monocyte-containing indices, of which AISI is a representative example, as inexpensive, immediately available candidates that merit formal evaluation in this population. Given the single-center design, the modest sample size and the ethnically homogeneous (Romanian, predominantly Caucasian) cohort, these results should be regarded as locally derived and hypothesis-generating; multicenter, prospective external validation in case-mix-diverse populations is required before any generalization to elderly ICU sepsis at large.

## Figures and Tables

**Table 1:** Baseline characteristics of the elderly sepsis cohort, stratified by 30-day mortality.

Variable	Overall	Survivors (*n* = 94)	Non-Survivors (*n* = 33)	*p*
Demographics				
Age, years	73 [69–79]	72 [68–79]	77 [73–79]	0.024
Male sex, *n* (%)	64 (50.4)	40 (42.6)	24 (72.7)	0.005
Severity at admission				
SOFA score	9 [7–10]	9 [7–10]	10 [7–11]	0.061
APACHE II	28 [26–30]	27 [24–29]	30 [29–31]	<0.001
Mechanical ventilation, *n* (%)	36 (28.3)	9 (9.6)	27 (81.8)	<0.001
LOS-ICU, days	6 [5–7]	6 [5–7]	7 [5–8]	0.036
LOS-Hospital, days	9 [7–11]	9 [8–11]	8 [6–9]	0.019
Comorbidities, *n* (%)				
Diabetes mellitus	31 (24.4)	23 (24.5)	8 (24.2)	1.000
Hypertension	59 (46.5)	47 (50.0)	12 (36.4)	0.251
Cardiovascular disease	72 (56.7)	52 (55.3)	20 (60.6)	0.747
Pulmonary disease	32 (25.2)	17 (18.1)	15 (45.5)	0.004
Malignancy (non-hem.)	34 (26.8)	16 (17.0)	18 (54.5)	<0.001
Renal disease	21 (16.5)	11 (11.7)	10 (30.3)	0.028
Hematology/chemistry				
Hemoglobin, g/dL	11.0 [9.1–12.6]	11.3 [9.6–12.7]	9.9 [8.2–12.3]	0.133
WBC, ×10^9^/L	9.5 [7.3–13.1]	9.3 [7.3–11.8]	15.8 [7.9–19.1]	<0.001
Neutrophils, ×10^9^/L	6.9 [4.9–10.4]	6.9 [4.9–9.7]	11.2 [4.9–16.1]	0.036
Lymphocytes, ×10^9^/L	1.16 [0.58–1.75]	1.20 [0.86–1.77]	0.90 [0.40–1.70]	0.094
Monocytes, ×10^9^/L	1.15 [0.56–1.38]	0.96 [0.47–1.33]	1.30 [0.87–2.08]	0.003
Platelets, ×10^9^/L	227 [164–286]	225 [172–278]	229 [152–302]	0.811
CRP, mg/L	74 [44–118]	74 [40–110]	65 [48–148]	0.337
Procalcitonin, ng/mL	0.86 [0.46–2.04]	0.84 [0.45–2.05]	1.02 [0.72–2.03]	0.534
Albumin, g/dL	3.10 [2.60–3.40]	3.30 [2.70–3.50]	2.80 [2.60–3.10]	0.003
LDH, U/L	254 [189–370]	224 [186–294]	367 [268–517]	<0.001
Creatinine, mg/dL	1.31 [0.79–1.92]	1.23 [0.71–1.87]	1.87 [1.40–3.12]	<0.001
Inflammatory indices				
NLR	5.69 [3.60–12.32]	5.33 [3.62–9.01]	11.00 [3.60–20.67]	0.023
PLR	190 [141–287]	167 [139–249]	238 [152–304]	0.096
MLR	0.82 [0.48–1.59]	0.69 [0.44–1.32]	1.46 [0.79–2.79]	<0.001
SII	1369 [864–2045]	1188 [828–1832]	1623 [1148–3401]	0.020
SIRI	6.54 [2.94–12.25]	4.79 [2.50–9.73]	12.07 [6.54–23.53]	<0.001
AISI	1293 [726–2732]	1009 [615–2082]	2914 [1405–3767]	<0.001
Number of comorbidities	3 [2–4]	2 [2–4]	5 [3–6]	<0.001

Continuous variables are presented as median [interquartile range]; categorical variables as *n* (%). Between-group *p*-values are derived from Mann–Whitney U-tests for continuous variables and Fisher’s exact/χ^2^ tests for categorical variables. APACHE II = Acute Physiology and Chronic Health Evaluation II; LOS = length of stay; WBC = white blood cells; CRP = C-reactive protein; LDH = lactate dehydrogenase; NLR = neutrophil-to-lymphocyte ratio; PLR = platelet-to-lymphocyte ratio; MLR = monocyte-to-lymphocyte ratio; SII = systemic immune-inflammation index; SIRI = systemic inflammation response index; AISI = aggregate index of systemic inflammation.

**Table 2:** AUROC for 30-day mortality, elderly sepsis cohort (*n* = 127, 33 deaths).

Marker	AUROC	95% CI	Optimal Cut-Off (Youden)	Sens	Spec	NPV
APACHE II	0.776	0.679–0.856	29	0.88	0.73	0.94
AISI	0.754	0.654–0.845	1304	0.82	0.62	0.91
SIRI	0.738	0.632–0.840	9.4	0.67	0.74	0.86
MLR	0.725	0.626–0.824	0.71	0.88	0.52	0.93
LDH (U/L)	0.717	0.599–0.828	—	—	—	—
WBC (×10^9^/L)	0.697	0.573–0.817	—	—	—	—
SII	0.637	0.526–0.744	2805	0.39	0.87	0.80
NLR	0.633	0.513–0.751	9.9	0.58	0.80	0.84
Age (years)	0.632	0.523–0.741	—	—	—	—
SOFA	0.609	0.490–0.720	—	—	—	—
PLR	0.598	0.468–0.723	227	0.64	0.70	0.85
CRP (mg/L)	0.556	0.440–0.668	—	—	—	—
PCT (ng/mL)	0.537	0.424–0.647	—	—	—	—

AUROC = area under the receiver operating characteristic curve; 95% CI from 2000 stratified bootstrap resamples. Sensitivity (Sens), specificity (Spec) and negative predictive value (NPV) are reported at the Youden-optimal cut-off. Cut-offs are not reported for variables not used as binary classifiers.

**Table 3:** Pairwise DeLong tests for AUROC differences.

Comparison	ΔAUROC	*z*	*p*
AISI vs. SII	+0.117	2.61	0.009
AISI vs. NLR	+0.121	2.14	0.032
AISI vs. PLR	+0.156	2.00	0.046
AISI vs. CRP	+0.197	2.81	0.005
AISI vs. PCT	+0.217	2.88	0.004
AISI vs. SIRI	+0.015	0.60	0.55
AISI vs. MLR	+0.029	0.72	0.47
AISI vs. APACHE II	−0.023	−0.33	0.74
SIRI vs. SII	+0.102	2.26	0.024
SIRI vs. NLR	+0.105	2.59	0.010
APACHE II vs. SII	+0.140	1.87	0.061
APACHE II vs. NLR	+0.143	1.72	0.086
APACHE II vs. PLR	+0.179	2.45	0.014
APACHE II vs. AISI	+0.023	0.33	0.74

Pairwise comparisons of AUROC using the DeLong test (Sun & Xu covariance method). Positive ΔAUROC indicates the first marker is superior. Selected comparisons of clinical and methodological interest are shown.

**Table 4:** Multivariable logistic regression for 30-day mortality. Each row represents a separate model containing the inflammatory marker, APACHE II and age (all standardized; markers log-transformed).

Marker (log-z)	aOR Marker (95% CI)	*p* Marker	aOR APACHE	*p* APACHE	aOR Age	*p* Age
AISI	2.80 (1.54–5.08)	0.001	2.37	0.004	1.44	0.117
SIRI	2.65 (1.51–4.62)	0.001	2.52	0.002	1.42	0.134
MLR	2.64 (1.50–4.64)	0.001	2.53	0.002	1.49	0.082
LDH	2.01 (1.28–3.14)	0.002	2.74	0.001	1.86	0.009
NLR	1.61 (1.05–2.46)	0.028	2.59	0.002	1.53	0.050
SII	1.54 (0.99–2.39)	0.058	2.50	0.003	1.56	0.039
PLR	1.17 (0.76–1.78)	0.482	2.55	0.002	1.59	0.033
Albumin (linear)	0.92 (0.60–1.42)	0.710	2.57	0.001	1.55	0.044

aOR = adjusted odds ratio per 1 standard deviation of the standardized predictor. All inflammatory markers were natural-log-transformed before standardization; APACHE II and age were standardized in their original scale. Each row is a separate three-predictor multivariable logistic regression model. The same APACHE II and age effects vary slightly across models because of mild collinearity with the marker.

**Table 5:** Reclassification metrics for the addition of each inflammatory marker to APACHE II.

Added Marker	AUROC Combined	ΔAUROC	IDI	*p* IDI	cNRI	*p* cNRI
AISI	0.834	+0.058	0.139	<0.001	0.671	<0.001
SIRI	0.827	+0.051	0.134	<0.001	0.567	0.005
MLR	0.814	+0.037	0.120	<0.001	0.571	0.005
NLR	0.811	+0.035	0.051	0.027	0.507	0.012
SII	0.801	+0.025	0.039	0.025	0.137	0.500

Reference model: APACHE II alone (AUROC 0.776, Brier 0.176, Hosmer-Lemeshow χ^2^ = 40.4, *p* < 0.001 for mis-calibration). IDI = integrated discrimination improvement; cNRI = continuous net reclassification improvement. The DeLong *p*-value for ΔAUROC of any marker added to APACHE II did not reach 0.05. IDI and cNRI are more sensitive to incremental information than the ΔAUROC test, but they can appear inflated in small samples and do not in themselves demonstrate clinical utility; they are therefore presented here as supportive, exploratory evidence rather than as confirmation of incremental discrimination.

## Data Availability

The datasets generated and analyzed during the current study are not publicly available, as they are part of an ongoing doctoral thesis, but are available from the corresponding author upon reasonable request.

## Supplementary Materials

The following supporting information can be downloaded at https://www.mdpi.com/article/10.3390/jcm15124799/s1. Table S1: Sensitivity analyses; Table S2: Operational characteristics of AISI at alternative cut-offs; Table S3: Spearman rank correlations between hematological inflammatory indices, APACHE II and LDH; Table S4: Descriptive characteristics of infection-source subgroups; Table S5: Comparison of candidate multivariable prediction models; Table S6: Net benefit at selected threshold probabilities from decision curve analysis; Figure S1: Spearman correlation heatmap; Figure S2: Discrimination of inflammatory indices and APACHE II stratified by mechanical ventilation status.

## Abbreviations

The following abbreviations are used in this manuscript:

AISI	Aggregate Index of Systemic Inflammation
aOR	Adjusted odds ratio
APACHE II	Acute Physiology and Chronic Health Evaluation II
APS III	Acute Physiology Score III
ARDS	Acute respiratory distress syndrome
AUC	Area under the curve
AUROC	Area under the receiver operating characteristic curve
BSI	Bloodstream infection
CBC	Complete blood count
CD8	Cluster of differentiation 8
CD14	Cluster of differentiation 14
CD16	Cluster of differentiation 16
CD45RA	Cluster of differentiation 45RA
CCR7	C-C chemokine receptor type 7
95% CI	95% confidence interval
cNRI	Continuous net reclassification improvement
CRP	C-reactive protein
DIC	Disseminated intravascular coagulation
FDA	U.S. Food and Drug Administration
GAM	Generalized additive model
HL	Hosmer-Lemeshow
HLA-DR	Human leukocyte antigen–DR isotype
HR	Hazard ratio
ICU	Intensive care unit
IDI	Integrated discrimination improvement
IL-1β	Interleukin-1 beta
IL-6	Interleukin-6
IQR	Interquartile range
LDH	Lactate dehydrogenase
LOS	Length of stay
LOWESS	Locally weighted scatterplot smoothing
MDW	Monocyte distribution width
MIMIC-IV	Medical Information Mart for Intensive Care IV
MLR	Monocyte-to-Lymphocyte Ratio
MPV	Mean platelet volume
NLR	Neutrophil-to-Lymphocyte Ratio
NPV	Negative predictive value
OR	Odds ratio
PCT	Procalcitonin
PIV	Pan-immune-inflammation value
PLR	Platelet-to-Lymphocyte Ratio
PPV	Positive predictive value
ROC	Receiver operating characteristic
SAPS II	Simplified Acute Physiology Score II
SD	Standard deviation
Sepsis-3	Third International Consensus Definitions for Sepsis and Septic Shock
SII	Systemic Immune-Inflammation Index
SIRI	Systemic Inflammation Response Index
SOFA	Sequential Organ Failure Assessment
STROBE	Strengthening the Reporting of Observational Studies in Epidemiology
TNF-α	Tumor necrosis factor alpha
TRIPOD	Transparent Reporting of a multivariable prediction model for Individual Prognosis or Diagnosis
VLBW	Very low birth weight
WBC	White blood cell (count)
