# Supplementary material for: Monocyte-Containing Inflammatory Indices Show Stronger Association with 30-Day Mortality than the Systemic Immune-Inflammation Index in Elderly Sepsis: A Single-Center Retrospective Observational Cohort Study
Source: J Clin Med. 2026 Jun 20;15(12):4799. doi: 10.3390/jcm15124799 (PMC13301268; doi:10.3390/jcm15124799)
Supplement: Supplementary file 1 [file jcm-15-04799-s001.zip › jcm-4378541-supplementary.pdf]

Supplementary Materials

Monocyte-Containing Inflammatory Indices Outperform the Systemic Immune-Inflammation Index for 30-Day Mortality Prediction in Elderly Sepsis

Irimie A.I., Dinescu S.N., Novac M.B., Vasile R.C., Rotaru-Zăvăleanu A.-D., Ruscu M.A., Radu L.

Journal of Clinical Medicine, 2026

Supplementary Table S1. Sensitivity analyses

Table S1. Adjusted odds ratios for log-transformed, standardised hematological inflammatory indices across alternative model specifications. Primary model adjusts for APACHE II and age (both standardised). All odds ratios are expressed per 1 standard deviation increase of the log-transformed index. Sample sizes and event counts reflect the primary analytic cohort of patients aged ≥ 65 years (n = 127, 33 deaths) except where noted. AUROC values for the mechanical ventilation subgroup analyses are descriptive only because the small number of events (6 in the non-ventilated subgroup) precludes meaningful multivariable adjustment.

Sensitivity analysis	n	Events	log-AISI aOR (95% CI)	p	log-SII aOR (95% CI)	p
Primary model (APACHE II + age)	127	33	**2.80 (1.54–5.08)**	0.001	1.54 (0.99–2.39)	0.058
+ Sex added (4-predictor)	127	33	2.68 (1.49–4.80)	0.001	1.54 (0.98–2.43)	0.061
+ LDH added (4-predictor)	127	33	2.38 (1.28–4.42)	0.006	1.30 (0.81–2.07)	0.272
Excluding active malignancy	93	15	2.54 (1.13–5.70)	0.024	—	—
Ventilated subgroup (AUROC, descriptive)	36	27	0.835 (0.69–0.96)	—	0.728 (0.53–0.90)	—
Non-ventilated subgroup (AUROC, descriptive)	91	6	0.706 (0.55–0.85)	—	0.527 (0.36–0.68)	—

Notes: aOR = adjusted odds ratio per 1-SD increase of the log-transformed, standardised index. The primary model adjusts for APACHE II and age (both standardised). Coefficients and 95% confidence intervals are from multivariable logistic regression. For the mechanical ventilation subgroup analyses, AUROC values with 2,000-bootstrap 95% confidence intervals are reported because event counts (especially 6 deaths in the non-ventilated subgroup) preclude reliable multivariable adjustment. The non-ventilated subgroup serves as a falsification check: NLR fell to AUROC 0.43, providing direct evidence that the platelet- and lymphocyte-driven indices do not retain prognostic signal once severe organ dysfunction is removed from the case mix, whereas AISI retained discrimination of 0.71. SII analyses in the malignancy-excluded subgroup are omitted because SII was non-significant in the primary model and the sub-analysis provided no additional information.

Supplementary Table S2. AISI at alternative cut-offs

Table S2. Operational characteristics of AISI at alternative cut-offs for 30-day mortality in elderly ICU sepsis (n = 127, 33 deaths). The Youden-optimal cut-off of 1,304 is highlighted (shaded). PPV, NPV, LR+ and LR− are computed using the cohort prevalence of 26.0%.

AISI cut-off	Sensitivity	Specificity	PPV	NPV	LR+	LR−
500	0.94	0.20	0.29	0.90	1.18	0.30
800	0.88	0.40	0.34	0.90	1.48	0.30
1000	0.85	0.49	0.37	0.90	1.66	0.31
**1304 (Youden)**	**0.82**	**0.62**	**0.43**	**0.91**	**2.14**	**0.29**
1500	0.73	0.64	0.41	0.87	2.01	0.43
2000	0.61	0.72	0.43	0.84	2.19	0.54
2500	0.55	0.83	0.53	0.84	3.20	0.55
3000	0.39	0.86	0.50	0.80	2.85	0.70

Notes: AISI = aggregate index of systemic inflammation; PPV = positive predictive value; NPV = negative predictive value; LR+ = positive likelihood ratio; LR− = negative likelihood ratio. The Youden cut-off (1,304) provides the best simultaneous trade-off between sensitivity and specificity but, as noted in Section 4.4 of the main manuscript, is derived and evaluated in the same sample and therefore requires prospective external validation. The 90+% negative predictive value across cut-offs from 500 to 1,304 supports use of low admission AISI as a safety-net 'rule-out' marker, with the caveat that PPV remains modest (0.29–0.43) at clinically practical cut-offs, consistent with the moderate AUROC of any single biomarker in this setting.

Supplementary Table S3. Spearman correlations

Table S3. Spearman rank correlations between hematological inflammatory indices, APACHE II score and lactate dehydrogenase in the elderly ICU sepsis cohort (n = 127). Coefficients ≥ 0.80 are shown in bold to highlight indices with substantial overlap in information content. Note the near-zero correlation between APACHE II and any inflammatory index, confirming that the combined model captures complementary rather than redundant prognostic information.

	NLR	PLR	MLR	SII	SIRI	AISI	APACHE	LDH
**NLR**	1.00							
**PLR**	0.44	1.00						
**MLR**	0.52	0.37	1.00					
**SII**	**0.86**	0.65	0.42	1.00				
**SIRI**	0.75	0.22	**0.85**	0.64	1.00			
**AISI**	0.61	0.32	**0.81**	0.68	**0.92**	1.00		
**APACHE**	−0.03	0.08	0.12	−0.01	0.10	0.14	1.00	
**LDH**	0.36	0.11	0.30	0.23	0.36	0.24	−0.06	1.00

Notes: Spearman rank correlation coefficients. APACHE = Acute Physiology and Chronic Health Evaluation II score; LDH = lactate dehydrogenase. The very high correlation between SIRI and AISI (ρ = 0.92) and between MLR and AISI (ρ = 0.81) reflects their shared mathematical structure: SIRI and AISI both incorporate neutrophils × monocytes / lymphocytes, with AISI multiplying further by platelets; MLR is the monocyte–lymphocyte ratio that anchors both. This redundancy explains the parallel directions of effect across these three monocyte-containing indices and supports presenting AISI as the primary candidate while reporting SIRI and MLR as complementary confirmations rather than truly independent predictors. The near-zero correlation between APACHE II and any haematological index (|ρ| ≤ 0.14) confirms that the combined APACHE II + AISI model captures genuinely complementary prognostic information, which is the mechanistic basis for the substantial cNRI and IDI improvements reported in Section 3.5.

Supplementary Table S4. Infection-source subgroups

Table S4. Descriptive characteristics of infection-source subgroups in the elderly cohort (n = 127). Subgroup sample sizes preclude inferential comparison; medians are reported to illustrate the consistency of the AISI elevation in non-survivors across sources of infection.

Infection source	n	Deaths (%)	Median AISI (overall)	Median AISI (non-survivors)	Median AISI (survivors)
Respiratory	15	6 (40.0%)	1,835	2,397	1,505
Gastrointestinal	35	9 (25.7%)	1,293	1,767	1,171
Urogenital	29	11 (37.9%)	1,827	3,767	820
Bone/soft tissue	7	0 (0%)	1,145	—	1,145
Other / unknown	6	1 (16.7%)	888	11,131	766

Notes: Sources of infection were not mutually exclusive; the totals therefore do not sum to 127. Patients with multiple potential sources were counted in each relevant category. Medians of AISI in non-survivors are consistently higher than in survivors across all subgroups for which both groups contained patients, supporting the source-independent nature of the AISI–mortality association. The bone/soft-tissue subgroup (n = 7) contained no deaths and is presented for descriptive completeness only. The single extreme value in the 'Other / unknown' non-survivor subgroup (AISI = 11,131) represents a single septicaemic patient and should not be over-interpreted as a subgroup pattern.

Supplementary Table S5. Multi-marker model comparison

Table S5. Comparison of candidate multivariable prediction models for 30-day mortality. AUROC values are accompanied by 2,000-bootstrap 95% confidence intervals. The pre-specified primary model (APACHE II + AISI) is shown in bold for reference.

Model	AUROC	95% CI	Brier score
APACHE II only	0.776	(0.676–0.856)	0.176
**APACHE II + AISI (primary)**	**0.834**	**(0.757–0.900)**	**0.148**
APACHE II + AISI + LDH	0.845	(0.773–0.905)	0.144
AISI + LDH + Albumin + age	0.788	(0.702–0.869)	0.155
AISI + LDH + age (3-predictor, APACHE-free)	0.778	(0.689–0.859)	0.156

Notes: The marginal gain from adding LDH to APACHE II + AISI (ΔAUROC +0.011) does not justify the additional predictor under the available events-per-variable budget (33 events / 3 predictors ≈ 11 EPV, consistent with the canonical heuristic). Notably, an APACHE-free model based on widely available laboratory variables (AISI + LDH + age) achieves AUROC 0.778, approaching APACHE II alone (0.776). This suggests that the APACHE II + AISI combination is the most parsimonious model that captures the available prognostic information; further additions are unlikely to be cost-effective in our sample size, and a fully APACHE-free alternative could be considered when APACHE II cannot be calculated routinely. External validation in larger cohorts is required to determine whether AISI + LDH + age can fully replace APACHE II in this population.

Supplementary Table S6. Net benefit at selected threshold probabilities

Table S6. Net benefit at selected threshold probabilities of 30-day mortality, derived from decision curve analysis (Figure 6). Δ Net Benefit = APACHE II + AISI minus APACHE II alone. Positive ΔNB indicates that the combined model identifies additional true positives without increasing false positives at the given threshold.

pt (%)	APACHE II + AISI	APACHE II alone	APACHE II + SII	Treat all	Δ NB (AISI vs APACHE)
**10%**	0.207	0.192	0.193	0.184	**+0.015**
**20%**	0.177	0.112	0.137	0.080	**+0.065**
**30%**	0.117	0.092	0.097	-0.057	**+0.026**
**40%**	0.067	-0.002	0.027	-0.233	**+0.069**
**50%**	0.023	-0.016	0.010	-0.480	**+0.039**

Notes: pt = decision threshold probability of 30-day mortality. Net benefit is on the scale of the outcome: e.g., a ΔNB of +0.065 at pt = 20% corresponds to 6.5 additional true high-risk patients correctly identified per 100 elderly ICU admissions when AISI is added to APACHE II, without any increase in false-positive triage. The 'treat all' net benefit becomes increasingly negative at higher thresholds, reflecting the cost of universal high-intensity intervention when most patients would not benefit. APACHE II alone becomes inferior to 'treat none' (net benefit < 0) at thresholds ≥ 40%, whereas APACHE II + AISI retains positive net benefit up to pt ≈ 55%.

Supplementary Figure S1. Spearman correlation heatmap

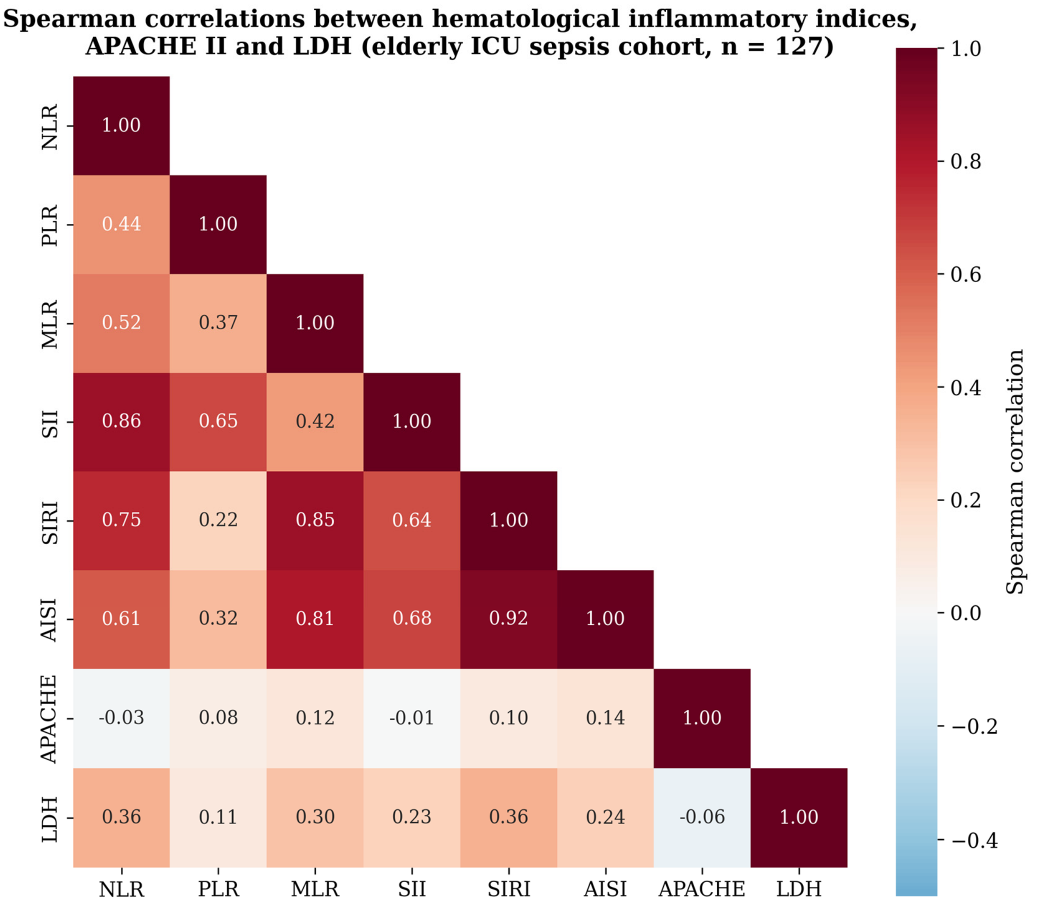

Figure S1. Spearman rank correlations between hematological inflammatory indices, APACHE II and lactate dehydrogenase in the elderly ICU sepsis cohort (n = 127). The heatmap visualises the same coefficients reported in Table S3. The block of high correlations among SIRI, AISI and MLR (lower-right quadrant of the inflammatory index block) reflects their shared mathematical structure built on the neutrophil × monocyte / lymphocyte core, while APACHE II shows near-zero correlation with all inflammatory markers, providing direct visual support for the complementarity that underlies the APACHE II + AISI combined model.

Supplementary Figure S2. Mechanical ventilation subgroup

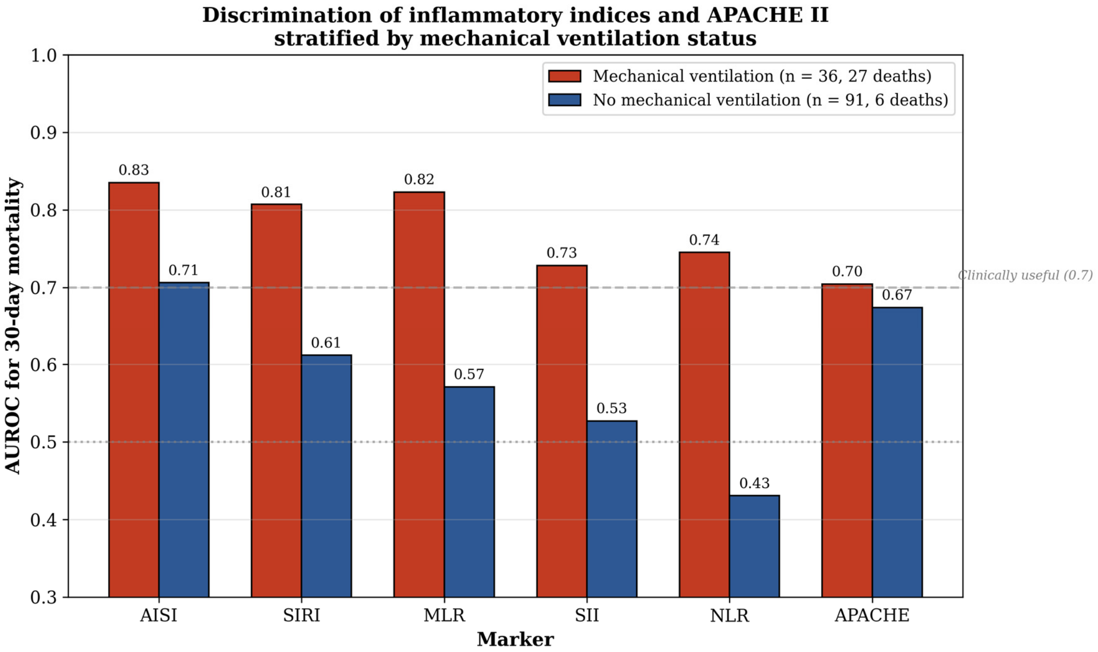

Figure S2. Discrimination (AUROC for 30-day mortality) of inflammatory indices and APACHE II stratified by mechanical ventilation status. In ventilated patients (red bars; n = 36, 27 deaths), AISI, SIRI and MLR all achieve AUROC > 0.80 and outperform APACHE II (0.70). In non-ventilated patients (blue bars; n = 91, 6 deaths), AISI alone retains discrimination above 0.70, while SII falls to 0.53 and NLR to 0.43 (below the chance line of 0.50, equivalent to inverse association). The differential between subgroups is largest for the platelet- and lymphocyte-driven indices (SII, NLR), supporting the interpretation that these markers carry prognostic signal only in the most severely affected sub-population, whereas monocyte-containing indices retain stratification value across the full cohort. The non-ventilated subgroup result (AISI AUROC 0.71 with 6 events) should be interpreted with caution given the very low event count, but provides a useful sensitivity check.
